# Mortality of St. Louis

**Published:** 1854-04

**Authors:** 


MORTALITY OF ST. LOUIS.—Dr. ENGLEMANN closes an elaborate paper in the *St. Louis Medical and Surgical Journal*, on this subject with the following remarks:

"In examining these tables, it must be borne in mind that it is not only the deaths that take place among our resident population, which are recorded there, but that the number is largely increased by the deaths occurring among the floating population, not included in the census tables, and the numerous travelers and immigrants, who not rarely arrive here in the last stage of sickness, only to people our burying places. I assume the population of the city and suburbs, in 1852, to have been 100,000, and in 1853, 110,000, which numbers however, are hardly large enough to include our floating population. The last column in the tables is based on this calculation. It results from this column, that in 1852, which was the last of our four cholera years, 48 died among 1,000, and in 1853, only 35; that from June to September, 1852, the number of deaths ranged from 4 to nearly 9 in 1,000, in one month, but that in 1853, the number never reached 4 in 1,000.

In both years the number of deaths of white females was 40, and of white males, 60 per cent., which is owing partly to the greater exposure and consequent sickness men are subjected to, but partly, also, to the absolutely much larger number of males in our community.

In 1852, the number of deaths of white chiidren of five years and under, was, in proportion, smaller than in 1853; in the former year it was 45, in the latter 53 per cent, The cause is to be found in the fact, that cholera attacked and destroyed fewer children, in proportion, than adults; we find the mortality among children in the summer months, when cholera prevailed, not to have been much more than one-third of the whole number of deaths. It is further to be found in the lower degree of heat which prevailed in the summer of 1852, and in the absence of fatal infantile epidemics In 1853, on the contrary, scarlatina of a very dangerous character, was prevalent for the four or six first months, almost exclusively among children; the proportion of infantile mortality, from January to April, was 52 per cent. In the months of June, and principally August, the heat of the weather found its easiest victims among young children, and we see the proportion, accordingly, much greater—in June, July and August together almost 60 per cent.

In the statistical investigation, published in this journal, in May last, p. 227, etc., I have already attempted to show the influence of atmospherical agents on the health of the summer and fall of 1852. It now remains to bring those tables down to the end of last year, (1853,) and by them either to confirm or to modify the results arrived at.

The per centage of sickness (see May number, page 228,) I find, for July 8; for August 12, and for September 13. The per centage of mortality was, in July 10, in August 11, and in September 11. The quantity of rain in May, June and July was nearly 11 inches, and in August and September a little over 10 inches. The mean temperature of June (78 degrees,) was higher than I ever observed it in that month; that of July (75 degrees,) was lower than the average; that of August (76 degrees,) and September (69 degrees,) is about the average temperature for both these months. These meteorological conditions would seem to indicate a sickly summer with a more healthy fall, but the low mean temperature of July evidently prevented much sickness in that month, which might have been expected from the want of humidity in that and the two preceding months. In August, we find a little more than the average sickliness, owing, no doubt, to the excessive heat of the second week of that month, (see above,) but less than the average mortality. In September sickliness as well as mortality were below the average."

